# Careers of an elite cohort of U.S. basic life science postdoctoral fellows and the influence of their mentor's citation record

**DOI:** 10.1186/1472-6920-10-80

**Published:** 2010-11-15

**Authors:** David G Levitt

**Affiliations:** 1 Department of Integrative Biology and Physiology, University of Minnesota, 6-125 Jackson Hall, 321 Church St. S. E., Minneapolis, MN 55455, USA

## Abstract

**Background:**

There is general agreement that the number of U.S. science PhDs being trained far exceeds the number of future academic positions. One suggested approach to this problem is to significantly reduce the number of PhD positions. A counter argument is that students are aware of the limited academic positions but have chosen a PhD track because it opens other, non-academic, opportunities. The latter view requires that students have objective information about what careers options will be available for them.

**Methods:**

The scientific careers of the 1992-94 cohort of NIH National Institute of General Medical Sciences (NIGMS) Kirchstein-NRSA F32 postdoctoral fellows (PD) was determined by following their publications (PubMed), grants (NIH and NSF), and faculty and industry positions through 2009. These basic life science PDs receive support through individual grant applications and represent the most successful class of NIH PDs as judged by academic careers and grants. The sex dependence of the career and grant success and the influence of the PD mentor's citation record were also determined

**Results:**

Of the 439 1992-94 NIGMS F32 fellows, the careers of 417 could be determined. Although females had significantly higher rates of dropping out of science (22% females, 9% males) there was no significant difference in the fraction of females that ended up as associate or full professors at research universities (22.8% females, 29.1% for males). More males then females ended up in industry (34% males, 22% females). Although there was no significant correlation between male grant success and their mentor's publication record (h index, citations, publications), there was a significant correlation for females. Females whose mentor's h index was in the top quartile were nearly 3 times as likely to receive a major grant as those whose mentors were in the bottom quartile (38.7% versus 13.3%).

**Conclusions:**

Sixteen years after starting their PD, only 9% of males had dropped out of science. More females (28%) have dropped out of science, primarily because fewer went into industry positions. The mentor's publication record does not affect the future grant success of males but it has a dramatic effect on female grant success.

## Background

In a recent front-page Miller-McCune article, Beryl Lieff Benderly presents a thorough review and discussion of the policies that have led to the current dismal science career prospects for U.S. PhDs. [1]. She proposes several dramatic changes in the current system, including limiting the number of PhDs trained, replacing the post-doctoral track with permanent career level investigators and limiting the number of foreign scientists in training. Recognition of the PhD glut is not new [2-5] and as early as 1998 a Research Council report recommended "restraint" in the growth of life science graduate students [5]. What is new is the hostility that has been aroused by the current economic crisis. Benderly describes a scientific establishment that is entrenched and selfish, "exploiting" the young scientists in a "Ponzi" scheme. The more than 204 online comments to this article provide an interesting cross-section of the attitudes of the scientists now entangled in this system. The majority of the comments are similar to those of an unemployed PhD that describes the current system as "indentured servitude". There were, however, some commenters that strongly differed with Benderly. The general view of this minority opinion was that the system was not broken, life sciences PhDs have very high employment rates (although not necessarily in academia) and the current post doctoral system and liberal admission of foreign students is essential for the success of American science. This minority view emphasized that the choice of a PhD career was made voluntarily with full knowledge of the limited career opportunities.

This last point raises the critical question of whether young scientists have accurate information and counseling about future career prospects. Ideally, an informed decision about whether to pursue a PhD should be based on reliable employment information. Just the simple demographics of the numbers of PhDs versus the number of future academic positions is insufficient because this does not distinguish among the broad range of disciplines and expertise represented by the PhDs and does not include the alternative professions (e.g. industry, government, etc.) that are available to basic scientists. The purpose of this study was to follow the careers of an elite cohort of PhDs that started their postdoctoral (PD) fellowship in 1992-94. They were recipients of the NIH Kirchstein-NRSA (F32) fellowship that is awarded based on individual research applications and represents the most successful class of NIH PD fellows based on their grant success and academic employment [6]. The focus was on applicants to the National Institutes of General Medical Science (NIGMS) which supports basic research "that is the foundation for disease diagnosis, treatment and prevention" and should represent the disciplines most relevant to academic and industry jobs. Although the career prospects of this1992-94 cohort were clearly much better than current PDs (see discussion), it is still useful to get some hard data on this recent group of outstanding PDs.

Another purpose of this study was to look at the correlation between the PDs grant success and their mentor's citation record. Although there have been a variety of publications with advice about how to choose a PD mentor [7-9], one aspect that is rarely mentioned is the importance of the mentor's numerical publication and/or citation record. In the current highly competitive environment, potential PDs should be aware of the factors that influence their future employment.

## Methods

All F32 NIGMS recipients whose fellowship started in the years 1992-94 were selected using the NIH RePORT site http://projectreporter.nih.gov/reporter.cfm. A small fraction had confusing names which did not allow definitive assignments of sex, grants, or careers. The PD's mentor was assumed to be the senior author on the publications of the PD from the fellowship institution during or shortly after the period of the fellowship. A small number of PDs had no such publications and therefore their mentor could not be determined. Table 1 lists the numbers for the various subgroups that were analyzed.

**Table 1:** Numbers of the NIGMS F32 1992-94 fellows (total = 439) whose sex, grants, careers and their mentor's publication record could be determined.

	Sex	Grants	Careers	Mentors
Males	280	279	268	249

Females	149	146	149	122

The PD's grant success was based on their success in obtaining grants from NIH (NIH RePORT) and NSF (NSF FastLane Award Search, https://www.fastlane.nsf.gov/a6/A6Start.htm) up to February, 2010. Grants were included only if they were clearly distinct from that of their mentor and, with a few exceptions, were from the period of 2000-9. A "major" grant was defined as: 1) 1 NIH R01 or P01; or 2) at least 2 NIH R21, R25, R43, R44 or P41; or 3) Large NSF grant (> $200,000). NIH K01, R03, R15, R29, R55 and SC3 were not considered "major". A "minor" grant was any grant that was not classified as "major". This classification was chosen because success in obtaining these "major" grants is usually evidence of an independent research program at a research institution.

The PD's current position was determined by following their grant (NSF or NIH) and publication record (using the "Institutions" filter of the ISI Web of Knowledge site to distinguish confusing names). Some non-scientific careers were followed through Google searches. From these records, the most recent employment site was determined and, if available, the details of the position were determined from a web search of this employment site. For those employed at academic institutions, the institutional site was checked and the employment position verified as of February, 2010. Because biomedical companies do not provide detailed information about employees, it was not possible to characterize the scientist's position in more detail or to confirm that they were currently employed. Seven different career categories were used: 1) "No publications" (Pubmed) after 1999. This group was subdivided into those for whom an alternative career (journalism, law, etc.) could be determined from an internet search. These people are considered to be no longer active in scientific research. 2) "Industry", as indicated by the most recent publication (after 1999). 3) "College, not tenured" indicated by current position at college or non-research university with rank less than associate professor 4) "College tenured" as indicated by current associate or full professor rank. 5) "Research - not independent" as indicated by joint publications from research university or research institute and either no academic appointment or appointment less than associate professor or lab director. In most cases, these investigators have not received major individual grant funding. 6) "Research institute group leader" as indicated by current title at non-academic institute. This includes, e.g., NIH intramural or FDA research groups, Craig Venter institute, etc. These investigators almost always also received major research grants. 7) "University tenured" as indicated by associate or full professor appointment at a research university.

The mentor's citation record (including abstracts) was determined using the ISI search site with the "Timespan" for the publications limited to the years 1992-98 (representative of the period of the fellowship) and the "Institution" of the publications limited to the organization listed for the fellowship. In a few cases when it was determined that the mentor had changed positions during the fellowship period, more than one institution was included. Three measures of the scientific impact of the mentor were analyzed: total publications, total citations, and the h-index [10,11]. The h-index is equal to the number of papers with citation number >= h. All citations through February 2010 were included. The reported citations results were determined using the "Citation Report" option of the ISI Web of Knowledge web site. The mentors were divided into 4 bins with increasing citation index. The cut-off for each bin was selected so that there were approximately the same number of mentors in each bin (because of the discrete nature of the index, there were not exactly the same number).

The only statistical analysis used was the standard chi-square test of significance [12]. Values indicated as "not significant" (NS) had a p value of greater 0.1.

## Results

Table 2 lists the most recent employment of the male and female PDs as a fraction of the total number. There was no significant difference between the percent of males (29%) and females (23%) that achieved university tenure positions. The main factor distinguishing the male and female careers was that more males ended up in industry (34% males versus 22% females), while more females are out of scientific research as indicated by no scientific publications after 1999 (9% males versus 22% females). Also, significantly more females were in non-independent research positions.

**Table 2:** Number and percent of males (total = 268) and females (total = 149) with their most recent employment in the different categories.

Most Recent Position: 2000 - 2009	Male		Female	
No scientific pubs after 1999	24	8.96%	33	22.1% (p < .005)
No position found	20	7.46%	32	21.4% (p < .001)
Journalism, Law, etc.	4	1.49%	1	0.671% (NS)

Industry	91	33.9%	33	22.1% (p = .012)

College not tenured	4	1.49%	2	1.34% (NS)

College tenured	13	4.85%	4	2.68% (NS)

Researcher - not independent	45	16.8%	40	26.8 (p = .015)

Research institute group leader	12	4.47%	3	2% (NS)

University tenured	78	29.1%	34	22.8% (NS)

The p value indicates the significance difference between males and females (chi square).

**Figure 1:**
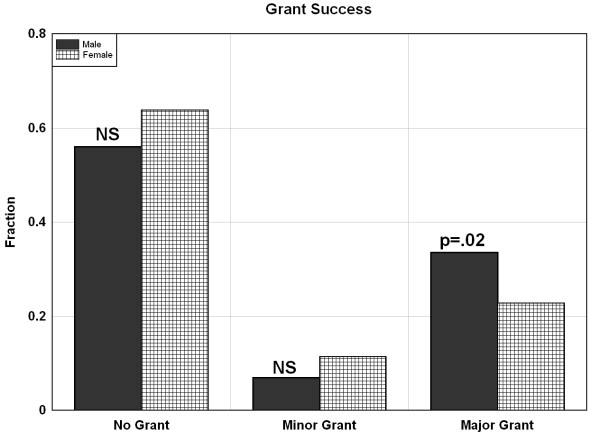
**Fraction of males (solid black) and females (cross-hatched) obtaining major, minor or no NIH or NSF grant**. The p value indicates the statistical significance of the difference between males and females (chi square).

Figure 1 shows the fraction of males and females that had either a) no grants; b) minor grants; or c) major grants in the period 1999-2009. There was no significant difference between males and females with no grants (56% males versus 63% females) or minor grants (6.9% males versus 1.14% females). However, significantly more males received major grants (33% males versus 23% females).

**Figure 2:**
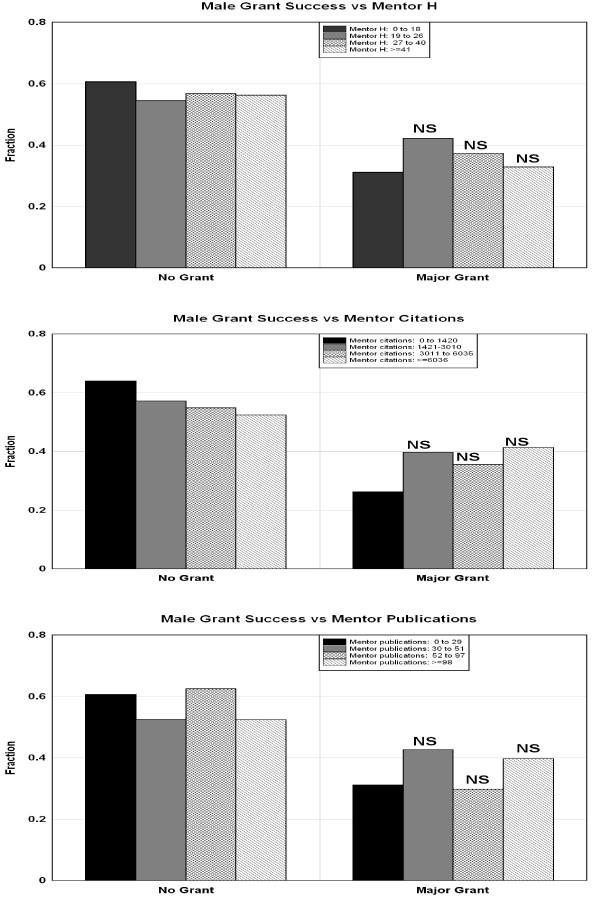
**Top panel: fraction of males that had a "major" grant (right) or no grant (left). Each bar indicates the fractions as a function of the mentor's h-index**. The statistical significance is for the difference between the fraction obtaining a major grant for the top 3 quartiles compared to the lowest quartile (chi square). Middle and bottom panels: similar plots for the mentor's total number of citations (middle) and publications (bottom).

**Figure 3:**
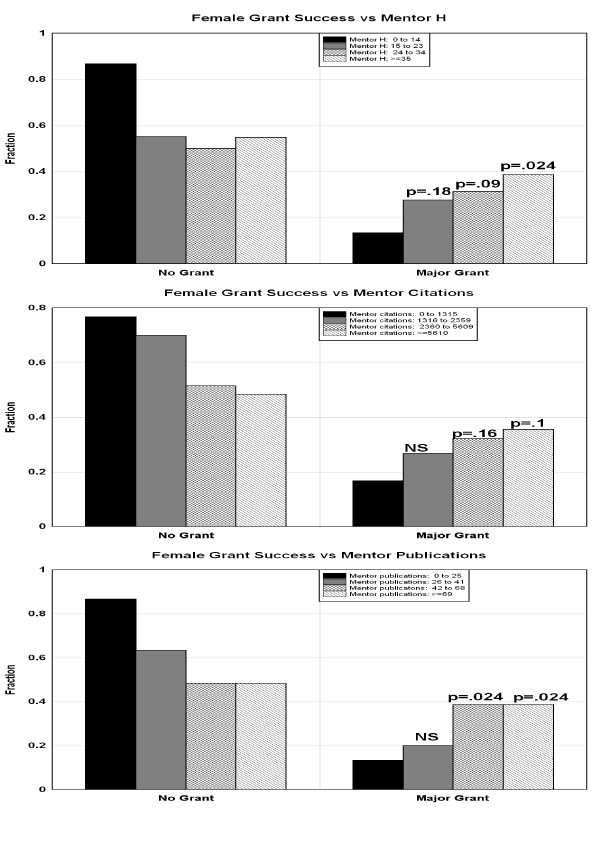
**Same as figure 2 for females**.

The top panel in figure 2 plots the fraction of the male PDs that did not (left panel) or did (right panel) receive an independent major grant as a function of the h index of their PD mentor. The p value indicates the significance of the difference between the fraction receiving a major grant in each of the top 3 categories versus the first (lowest) categories. There was no significant dependence of male grant success on the mentor's h-index. The middle and lower panels in figure 2 show similar plots as a function of the mentors total number of citations and total number of publications. Figure 3 shows a similar plot for females. Unlike males, the major grant success of females had a marked and statistically significant dependence on the mentors h-index and total number of publications. Females whose mentor's h-index were in the top quartile were nearly 3 times as likely to receive a major grant as those whose mentors were in the bottom quartile (38.7% versus 13.3%). The average h-index of the males' mentors was significantly (p = 0.013) higher than that of the females' mentors (30.2 males versus 26.2 females), indicating that females selected mentors with somewhat lower overall h-index. There was no significant difference in the average number of total citations or publications of the male and female mentors.

The correlation between the PD being "out of science" versus the mentor's citation record was also determined. There was no significant correlation for either males or females.

## Discussion

These results can be compared with those of the comprehensive NIH study "Career Achievements of National Research Service Award Postdoctoral Trainees and Fellows: 1975-2004" [6] which followed fellows that received the award in the period 1975- 1992. In the NIH study 35% of the F32 fellows received an "R01 equivalent" grant in the first 10 years after receipt of the doctorate, compared to the present study in which 30% (males and females) received an independent "major" grant any time up to 2009. Since the current classification of "major" grant should include any "R01 equivalent" grants and the current grant period is longer than that of the NIH study, this result indicates that a smaller percentage of the 1992-94 F32 fellows were successful in obtaining major grants compared to the earlier period.

In the current study the employment positions of all of the 1992-94 F32 fellows was obtained, while in the NIH study the employment positions of only a small subset of the total F32 pool was followed using the 1995 Survey of Doctorate Recipients (SDR). In the NIH study, 12 years after receiving the doctorate degree, 46% of the surveyed F32 fellows held associate or full professor positions at an academic research institution compared to 27% (male and females) in the current study, indicating a much tougher academic job market for the more recent PDs.

The NIH study looked at the regression parameters of career success for males and females for the entire Kirchstein PD group (F32 plus T32). Males were significantly more likely to obtain an R01 equivalent grant, similar to the results of this study. In the NIH study, a significantly higher fraction of females were employed at academic research institutions, but males were significantly more likely to be employed at the associate or full professor position. In the present study, although a higher fraction of males were associate or full professors; the difference was not significant. The NIH study did not look at the mentor's publication record, but it did look at regression parameters based on the NIH extramural funding ranking of the postdoctoral institution. It found a significant positive correlation between this ranking and both the fraction of PDs receiving an R01 and the total number of publications of the PDs.

The gender gap in academic research appointments has been the focus of many studies [13-15]. Recent analysis indicates that although there is no difference in male versus female grant success rates, a higher proportion of women tend to drop out of scientific research after the PD for lifestyle reasons [13,14]. This is supported by the current results where 22% of females are no longer publishing compared to 9% males. Surprisingly, despite this higher dropout rate, the difference in the fraction of male and female PDs that secured tenure track academic research positions was small and not statistically significant (29% males versus 23% females). The main difference between male and females careers is that a significantly smaller fraction of women ended up in industry (34% males versus 22% females). This suggests that women are more likely to opt out of a research career if the only option is industry.

The only previous investigation of the link between the mentor's citation record and the PDs career that could be found was a study that found no significant correlation between the mentor's h-index or number of publications and the subsequent total number of publication of the NIMH PD trainees at the University of Colorado from 1979 to 2004 [16]. A publication from the Zagreb University School of Medicine [17] found a strong short term correlation between the mentor's and the fellows publications. However this study does not exclude publications of the fellow while still with the mentor. In the results describe here, the PDs grant success was only included after the PD had become independent of the mentor. No previous analysis of the sex dependence of the correlation between the mentor and PD success could be found in a literature search. This new analysis finds a surprising sex dependence of this correlation. For males, there was no significant correlation between PD grant success and the mentor's publication record. However, for females there was a significant correlation with either the h-index or the total number of citations of the mentor. Females whose mentor's h-index were in the top quartile were nearly 3 times as likely to receive a major grant as those whose mentors were in the bottom quartile (38.7% versus 13.3%). This might suggest that there is some residual sexual discrimination in tenure track hiring, which can be overcome by a prestigious PD. This result requires further investigation. If confirmed, this represents a factor that should enter into a female's choice of a PD mentor.

One limitation of this analysis is that it focuses only on the careers of PDs that are either U.S. citizens or permanent residents, since this is a requirement of this NIH PD award. Of the 417 PDs whose careers could be traced, only 9 ended up at positions outside the U.S. (1 France, 1 China, 3 Canada, 3 Germany, 1 Israel).

With the average age that scientists are awarded their first independent NIH grant now at 42 [18], it is necessary to follow careers for many years in order to evaluate their outcome. Thus, of necessity, the subjects of this study began their PD training 16 to 18 years ago. This raises the obvious question of the relevance of this analysis for today's life science students. One approach to this question is to compare today's scientific career options to those of the 1992-94 PDs. The 1992-94 PD cohort had the advantage of two booms in scientific hiring: 1) the doubling of the NIH budget between 1998 and 2003; and 2) the growth in the biotech industry. This NIH budget doubling was accompanied by a 48% increase in total medical school Ph.D. faculty between 1993 and its peak in 2004 [19]. Academic employment has leveled off or even decreased since 2004 (there was a 1% decrease between 2004 and 2006 [19]). With the current hiring freeze at many medical schools [20], this flat or decreasing trend is likely to continue for several years. This disequilibria in academic employment produced by the NIH budget doubling is well recognized [2,4,21]. Less recognized is the major importance of industry in absorbing the PDs produced during this period. More of these 1992-94 F32 PDs ended up in industry than obtained research academic tenure positions. From 1994 - 2008, while the NIH budget grew by $17.5 billion ($10.4 to $27.9), the biomedical industry research budget (pharmaceutical, biotech and medical device) grew by $39.9 billion ($21 to $60.9) [22,23]. The recent surge in pharmaceutical layoffs [24] suggests that this growth has also leveled off. The big question is whether this recent flattening of academic and industry hiring is temporary or if it is a harbinger of a long term trend in U.S. economics.

## Conclusions

For this 1992-94 cohort the PD training provided a successful admission to a scientific career with 91% of males and 72% of females still actively employed in science related positions in 2009. A surprising finding is that the publication record of the PD mentor has a major effect on the future grant success of females but no significant effect on males. A major uncertainty is whether these results can be extrapolated to today's PDs.

## List of abbreviations

PD: postdoctoral fellow.

## Competing interests

The author declares that they have no competing interests.

## Authors' contributions

DGL was responsible for all aspects of this manuscript.

## Pre-publication history

The pre-publication history for this paper can be accessed here:

http://www.biomedcentral.com/1472-6920/10/80/prepub
